# External Validation and Feedback-Driven Improvement of the Diagnostic Model for Pediatric Cervical Lymphadenopathy

**DOI:** 10.3390/cancers18152407

**Published:** 2026-07-26

**Authors:** Rachel M. A. Vreugdenhil, Tammo P. A. Beishuizen, C. Michel Zwaan, Auke Beishuizen, Eline A. M. Zijtregtop

**Affiliations:** 1 Department of Pediatric Hematology and Oncology, Erasmus Medical Centre-Sophia Children’s Hospital, Wytemaweg 80, 3015 CN Rotterdam, The Netherlands; 2 Department of Pediatric Hemato-Oncology, Princess Máxima Center for Pediatric Oncology, Heidelberglaan 25, 3584 CS Utrecht, The Netherlands

**Keywords:** lymphoma, pediatric, children, cervical, lymphadenopathy, diagnostic model, non-Hodgkin lymphoma, Hodgkin lymphoma, diagnosis

## Abstract

**Background/Objectives**: Early identification of high-grade lymphoma in children presenting with cervical lymphadenopathy is essential to ensure timely referral while avoiding unnecessary invasive procedures. We have previously developed a diagnostic model to support referral decisions. The aim of this study was to validate this model in an independent cohort and improve it based on clinical feedback and new data. **Methods**: We included 255 pediatric patients with cervical lymphadenopathy, of whom 62.4% were diagnosed with malignancy. External validity was assessed by applying the original scoring model to the new dataset and calculating the sensitivity, specificity, and area under the curve (AUC). Thereafter, the model was updated by addressing overlapping predictors, and evaluating additional variables that were identified through univariate analysis. **Results**: The original model demonstrated stable performance in the new cohort, with sensitivity and specificity remaining within the previously reported 95% confidence intervals. No new clinically relevant predictors emerged beyond those already included in the original model. Model refinement resulted in a simplified model with 9 variables (reduced from 12), which improved clinical usability by reducing redundancy among predictors and facilitating implementation in routine practice. The revised model showed stable diagnostic accuracy with a sensitivity of 94% and specificity of 91% in the new dataset. **Conclusions**: Our updated diagnostic nine-factor model maintains high diagnostic performance while having a reduced number of variables. These findings support its use as a practical tool for early identification of children at risk of high-grade lymphoma.

## Simple Summary

Cervical lymphadenopathy is a common finding in children. Distinguishing between benign and malignant causes can be challenging, which may lead to unnecessary referrals or delayed diagnosis. In a previous study, we developed a diagnostic model to support early decision making in children with suspected lymphoma. In the present study, we validated this model in a new group of patients and improved it based on clinical feedback. The updated model uses fewer variables, making it easier to apply in daily practice, while maintaining high diagnostic accuracy. This approach may help clinicians identify children at risk of lymphoma more efficiently, support timely referral, and reduce unnecessary diagnostic procedures in those with benign disease.

## 1. Introduction

Cervical lymphadenopathy is a common clinical finding in children and adolescents. Usually, it is caused by benign conditions such as infections [1,2,3,4,5]. However, malignancy, specifically high-grade lymphomas like classical Hodgkin lymphoma (cHL) and non-Hodgkin lymphoma (NHL), can often not be ruled out [6,7]. For these high-grade lymphomas, early diagnosis is essential, as delays may lead to more widespread disease and increased toxicity from more intensive treatments [8,9]. Pediatricians therefore face a diagnostic dilemma: they need to identify children who need urgent referral to a pediatric oncologist while at the same time avoiding unnecessary invasive procedures, such as biopsies. Current clinical guidelines are often ambiguous, and the existing literature provides inconsistent data regarding predictive factors for lymphoma [10,11,12,13,14,15,16].

To resolve this problem, Zijtregtop et al. developed a 12-factor logistic regression machine learning diagnostic model to identify high-grade lymphoma [17]. The model consisted of various factors, including findings from physical examination; laboratory and imaging variables, such as elevated thymus and activation-regulated chemokine (TARC); presence of an enlarged mediastinum; and presence of >3 affected cervical lymph node regions. The model was highly sensitive and specific in detecting high-grade lymphoma in children with lymphadenopathy.

Since the paper by Zijtregtop et al., studies have shown similar results in univariate analyses [17]. In multivariate analyses, lymph node size and the presence of pathological lymph nodes on ultrasound were the most important predictors of lymphoma. However, none of these studies have developed a prediction model for use in a clinical setting [10,13,18,19,20,21].

The diagnostic model has not yet been implemented in clinical practice. External validation is essential to evaluate the reliability of the model. Therefore, a new cohort of patients was collected to test the model. Moreover, clinical feedback was used to refine, and where necessary, update the model to improve its accuracy and clinical applicability.

## 2. Materials and Methods

### 2.1. Study Design and Population

This retrospective, single-center study included patients referred to the Princess Máxima Center for Pediatric Oncology in Utrecht, the Netherlands. The Princess Máxima Center is a tertiary cancer center and patients were mainly referred by general pediatricians. Patients were included if they presented with cervical or supraclavicular lymphadenopathy with suspected lymphoma. Informed consent was obtained from all patients and/or their parents or guardians. Patients aged 0–18 years who were evaluated at the Princess Máxima Center between January 2022 and October 2025 were considered for inclusion. Patients with relapsed lymphoma or post-transplant lymphoproliferative disorders (PTLD) and patients with hereditary tumor-predisposition syndromes were excluded from the study. Furthermore, patients with nodular lymphocyte-predominant Hodgkin lymphoma (NLPHL) and other low-grade lymphomas were excluded from this study, in accordance with the methodology of the original diagnostic model [17]. The original study demonstrated that NLPHL could not reliably be distinguished from benign lymphadenopathy using the available predictors. Moreover, the primary aim of the model is to support the early identification and referral of children with high-grade lymphoma, for whom timely diagnosis is most critical [22]. Accordingly, the model was specifically developed for high-grade lymphoma risk stratification and should not be applied to detect or exclude NLPHL or other indolent lymphomas.

Data collection about the same predictive factors was performed in the same manner as described in the study of Zijtregtop et al. [17].

The study was approved by the Dutch Medical Research Ethical Committee Utrecht under trial number 21-073/C and number 16-739.

### 2.2. Feedback-Driven Model Improvement

Following publication of the original model, we received feedback from clinicians indicating that the model had a relatively high number of predictors and that several predictors had considerable conceptual overlap. Specifically, the variables ‘level V lymph node’ and ‘supraclavicular lymph node’ were frequently reported together in clinical practice, suggesting redundancy. A similar overlap was noted between ‘hilar/mediastinal involvement’ and ‘enlarged mediastinum’. Therefore, we checked for collinearity in the original dataset [17].

Clinicians also noted that the cut-off values used for laboratory parameters appeared arbitrary. In particular, the lactate dehydrogenase (LDH) threshold did not account for the known age-dependent variation in LDH levels, which are physiologically higher in younger children according to existing pediatric reference data [23,24].

### 2.3. Statistical Analysis

#### 2.3.1. Univariate Analysis

To determine differences between the malignant and the benign group, Fisher’s exact test was used for binary variables, and the Mann–Whitney U test was used for continuous variables. Laboratory values were analyzed both as continuous factors as well as binary variables using the cut-off point derived from reference ranges used at the Princess Máxima Center. A *p*-value of <0.01 was considered statistically significant.

The same statistical tests were used to examine differences between the old dataset (from Zijtregtop et al.) and the new dataset which was collected during this study [17].

Univariate analyses were performed with R, version 4.4.1 (R Foundation for Statistical Computing, Vienna, Austria).

#### 2.3.2. External Temporal Validation

To evaluate the performance of the previously developed diagnostic model in an independent cohort, the original scoring model was applied to the newly collected patient dataset. For each patient, the variables included in the initial model were extracted using the same definitions and cut-off values as described in the original publication. The total score was calculated according to the original weighting factors, and patients were classified using the previously established cut-off score of 27.5. All missing variables were marked as negative, assuming a diagnostic test is generally not performed when the suspicion of abnormalities is low, consistent with the methodology of the paper by Zijtregtop et al. [17]. To evaluate the potential impact of this approach, several sensitivity analyses were performed. First, the model was retrained using only patients with complete data. Second, missing values were treated as a separate predictor category. Third, missing values were randomly assigned as present or absent. Model performance was subsequently compared across these approaches using sensitivity and specificity.

Model performance in the new cohort was assessed by calculating sensitivity and specificity for identifying high-grade lymphoma and area under the curve (AUC). These results were compared with the performance reported in the previous article to determine the model’s generalizability. All analyses were performed using Python Programming software, version 3.12.10 (Python Software Foundation, Wilmington, DE, USA).

#### 2.3.3. Model Updating Procedures

Model revision was performed in a structured, stepwise manner. First, variables showing collinearity were identified using a correlation matrix. Each pair of overlapping variables was assessed through ablation studies by selectively removing one predictor and then evaluating changes in the performance of the model. This approach ensured that any reduction in predictor count would not adversely affect the sensitivity and specificity of the model. Second, the laboratory parameter LDH was redefined using an age-adjusted binary variable based on established pediatric reference values, replacing the previously fixed cut-off. Third, predictors with minimal contribution to the model, which were identified by a low weight factor, were evaluated for removal. Variables that did not affect the performance of the model were excluded. Finally, potential new predictors were identified in the univariate analysis of the new dataset. If predictors improved the performance of the model, we considered adding them to the model.

Model performance of the revised model was evaluated by calculating sensitivity, specificity and AUC. Moreover, the receiver operating characteristic (ROC) curve was visualized. The degree of calibration of the model was assessed by generating a calibration plot and calculating the Brier score.

## 3. Results

### 3.1. Study Population and Baseline Characteristics

**Figure 1:**
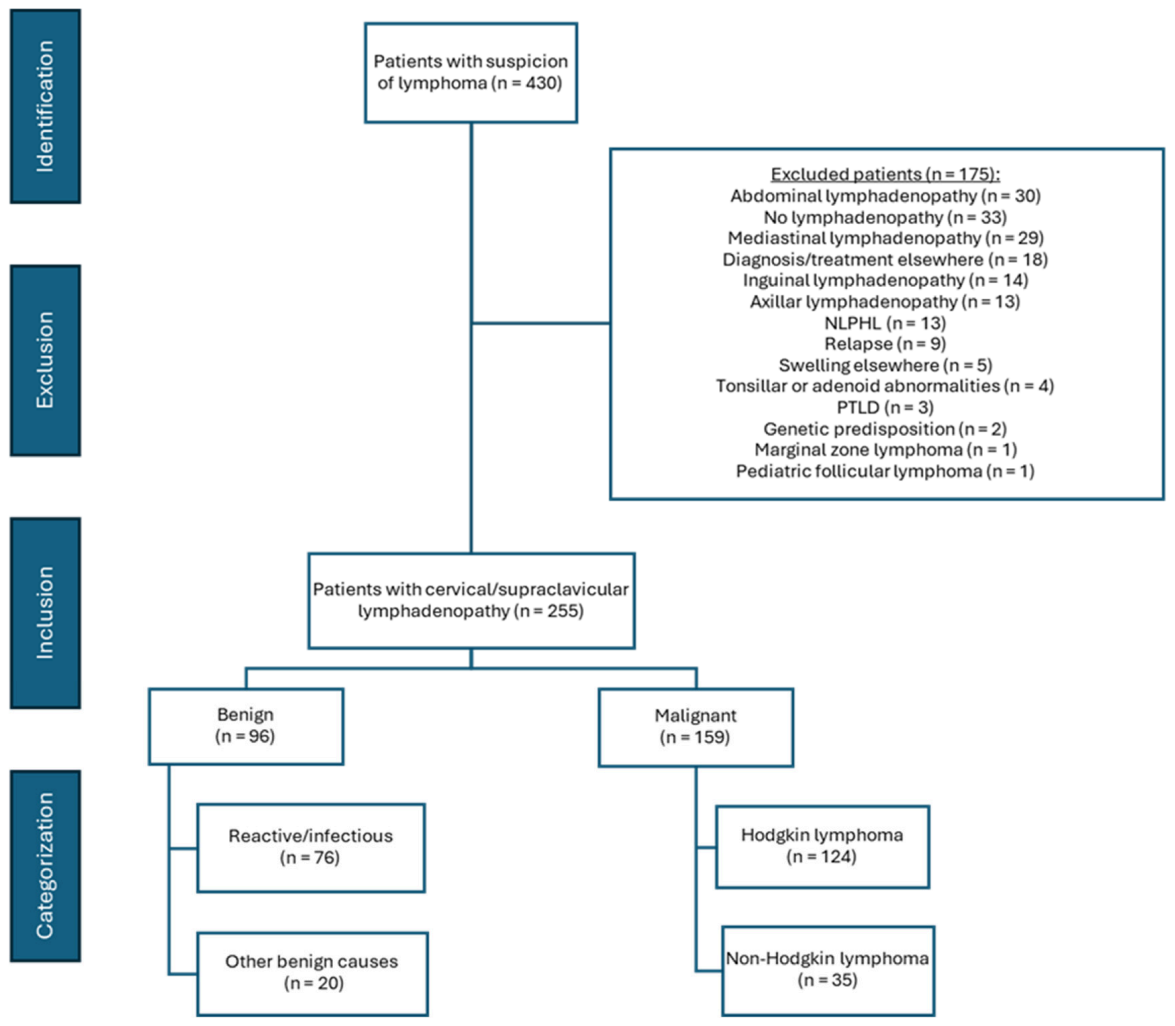
CONSORT flow diagram of study participants. Abbreviations: PTLD—post-transplant lymphoproliferative disorder; NLPHL—nodular lymphocyte-predominant Hodgkin lymphoma.

A total of 430 patients were referred, but after excluding patients as mentioned above, 255 patients with cervical lymphadenopathy were included in the study. Of these patients, 159 (62.4%) were diagnosed with a malignancy (see CONSORT flow diagram in Figure 1), consistent with the role of the Princess Máxima Center as a tertiary center. Not all cases were biopsied; an overview is given in Table S1.

Baseline characteristics of the old dataset (2018–2020) and the new dataset (2022–2025) are shown in Table S1. A more detailed comparison of both datasets is provided in Table S2. In summary, patients in the new dataset showed a lower prevalence of hepatosplenomegaly (*p* = 0.002), and more frequently demonstrated LDH values exceeding the age-adjusted thresholds used at the Princess Máxima Center (*p* = 0.006), as well as neutrophil counts > 6 × 10^3^/mm^3^ (*p* = 0.017). Aside from these differences, the datasets did not differ significantly.

### 3.2. Univariate Analysis Predictive Factors

Univariate analysis identified 15 predictive factors for high-grade lymphoma. The results of the univariate analysis are presented in Table 1. Due to the retrospective design of the study, certain data were missing, particularly regarding laboratory values. Details regarding the missing data can be found in Table 1 as well.

The results largely correspond to those of the previous article, although hepatosplenomegaly (*p* = 0.077) and presence of elevated LDH (*p* = 0.015) showed no significant difference between the benign and malignant group, in contrast to the findings of Zijtregtop et al. [17].

The findings of the univariate analysis were used to explore potential predictors and to evaluate consistency with the original model. Although LDH did not demonstrate a significant association in the benign-versus-malignant comparison, subgroup analyses showed that LDH levels differed significantly between NHL and both HL (*p* = 0.010) and benign cases (*p* = 0.005) (Table S3). This is consistent with previous findings, where LDH was specifically identified as a predictor for NHL rather than for lymphoma as a whole. In line with the methodology of the original study, LDH was therefore retained in the model due to its established clinical relevance and its demonstrated contribution to improving discrimination of NHL in multivariable analysis.

### 3.3. External Validation

When applied to the new dataset, the original scoring model demonstrated a sensitivity of 94% and a specificity of 91% for detecting high-grade lymphoma, using the same cut-off score as defined in the initial publication. The AUC was 0.98 (95% CI 0.96–0.99), indicating good discriminatory performance.

Compared to the original development cohort, sensitivity changed from 95% to 94%, while specificity changed from 88% to 91%. The number of false-negative classifications increased from two to nine, and the number of false-positive classifications changed from six to eight, in a total of 255 patients (versus 171 in the original cohort). The full clinical profile of all misclassified cases is provided in Table S5.

Sensitivity analyses demonstrated that model performance was largely unaffected by the method used to handle missing values (Table S6). Restricting the analysis to complete cases resulted in a sensitivity of 96% and specificity of 79%. When missing values were treated as a separate category, sensitivity and specificity were 94% and 86%, respectively. Random assignment of missing values yielded a sensitivity of 94% and specificity of 87%. Overall, these findings indicate that the model remained stable across different missing-data strategies.

### 3.4. Model Improvement

A correlation matrix showed significant overlap in the variables ‘level V lymph node’ and ‘supraclavicular lymph node’ and the variables ‘hilar/mediastinal involvement’ and ‘enlarged mediastinum’. Removing ‘enlarged mediastinum’ and ‘level V lymph node’ did not change sensitivity or specificity. Therefore, these variables were excluded from the model.

**Figure 2:**
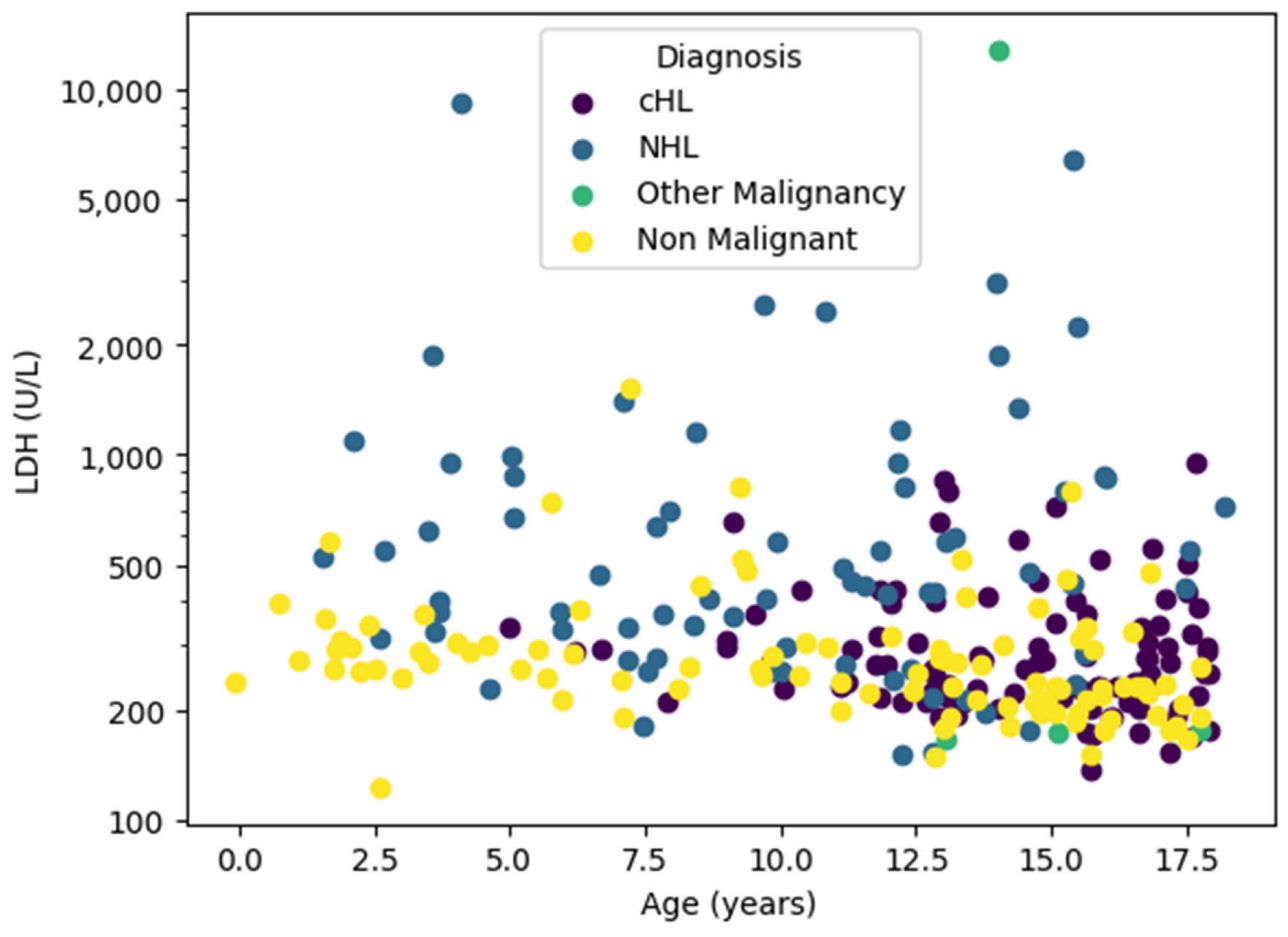
LDH distribution. Abbreviations: cHL—classical Hodgkin lymphoma; NHL—non-Hodgkin lymphoma; LDH – lactate dehydrogenase.

Examination of the original dataset demonstrated a clear correlation between LDH and age, indicating the need for age-specific interpretation. This correlation is demonstrated visually in Figure 2. Therefore, the fixed threshold ‘LDH > 260 U/L’ was replaced with a new binary variable (‘LDH > Princess Máxima center cut-off’), which uses age-adjusted normal values derived from reference ranges used at the Princess Máxima Center (Table S4).

The variable ‘neutrophils > 6 × 10^3^/mm^3^’ contributed minimally to the model, as reflected by its very low weight factor. Consistent with expectations, its exclusion did not alter sensitivity or specificity. Table S7 provides an overview of all variables considered for removal from the model and their impact on model performance.

**Figure 3:**
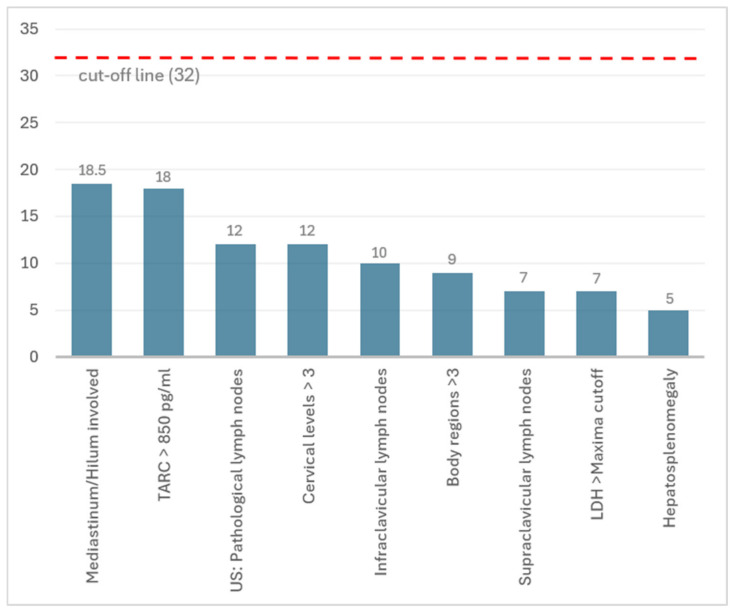
Factors included in the new model with their weighting factors. Abbreviations: TARC—thymus and activation-regulated chemokine; LDH—lactate dehydrogenase; US—ultrasound; Maxima—Princess Máxima Center.

Finally, based on the univariate analysis, we tried to add several new variables, such as age, size of the lymph node, and ESR. However, no variable improved the accuracy of the model. The final variables of the model, their definitions, and the weight factors are shown in Table 2. Additionally, a graphic representation of the final variables is depicted in Figure 3.

### 3.5. Performance of the Updated Model

After updating the diagnostic model, its performance was evaluated in both the original development dataset and the newly collected patient cohort. In the validation cohort of the original dataset, the updated model achieved a sensitivity of 95% and a specificity of 90%, which was comparable to the performance of the initial scoring model. The AUC for the updated model in the development cohort was 0.95 (95% CI 0.90–0.99). When applied to the new dataset, the updated model demonstrated a sensitivity of 94% and a specificity of 91%, which is exactly the same as the results of the original model. The AUC was 0,98 (95% CI 0.96–0.99).

**Figure 4:**
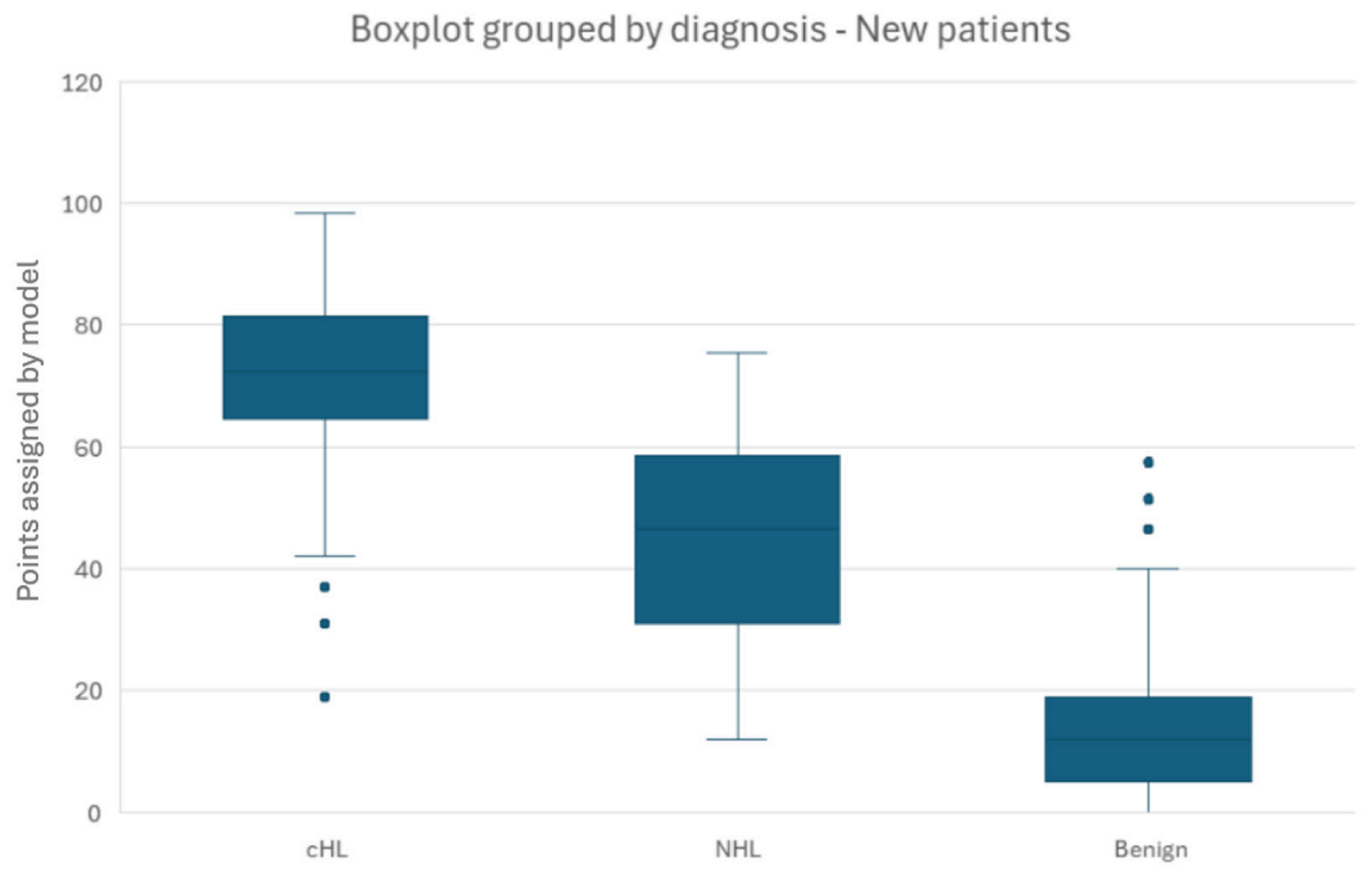
Boxplot—new patient cohort. Overview of scored points according to the revised model distributed across the three outcome groups. Abbreviations: cHL—classical Hodgkin lymphoma; NHL—non-Hodgkin lymphoma.

**Figure 5:**
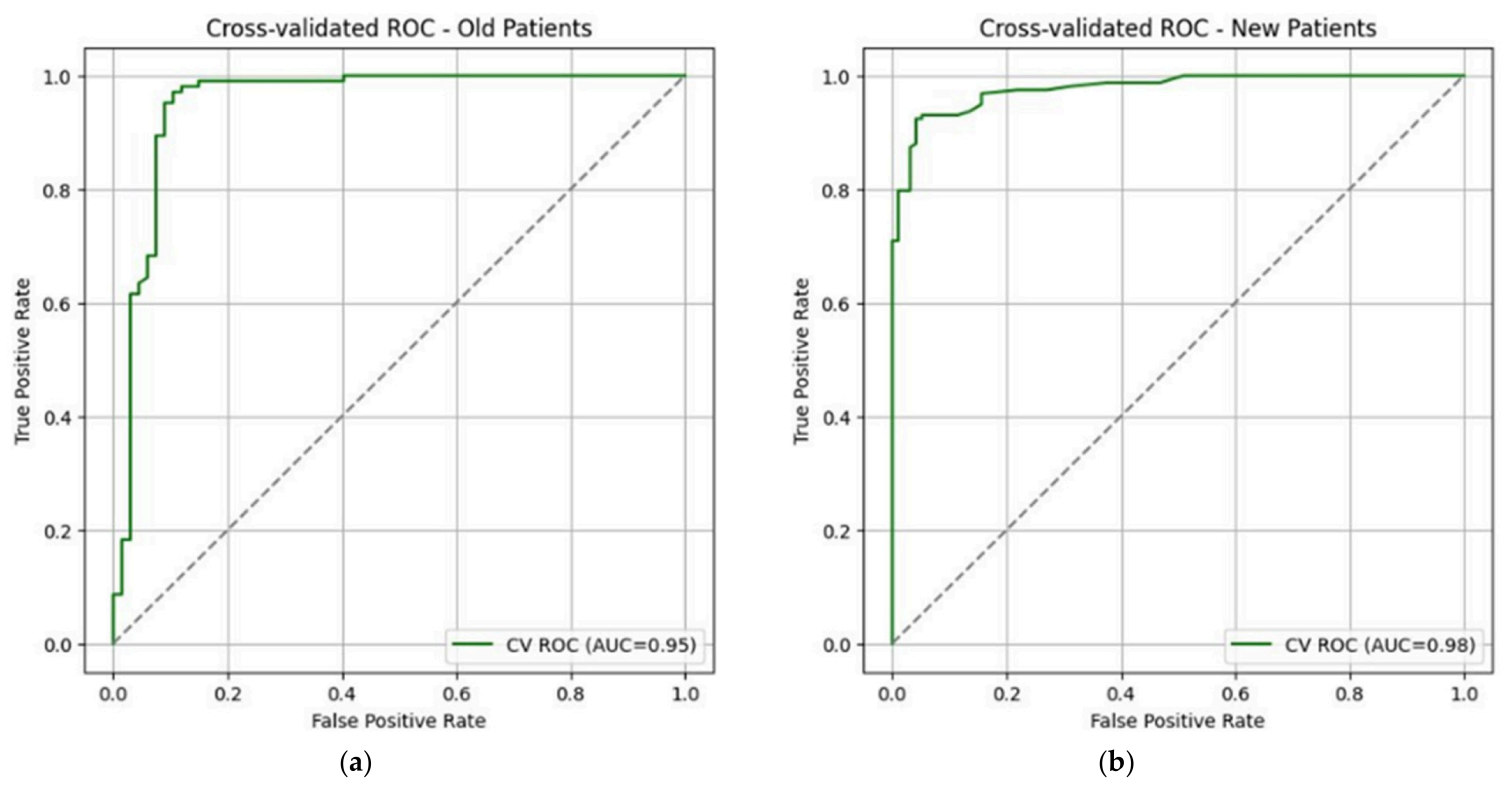
(**a**) ROC curve of the validation cohort of the original dataset; (**b**) ROC curve of the new dataset. Abbreviations: CV—cross validation; ROC—receiver operating characteristic.

Overall, the updated model showed consistent performance across both datasets. An overview of the scored points according to the new model is presented in Figure 4, and the ROC curves are presented in Figure 5. The performance of both the original 12-variable model and the revised 9-variable model is summarized in Table S8.

**Figure 6:**
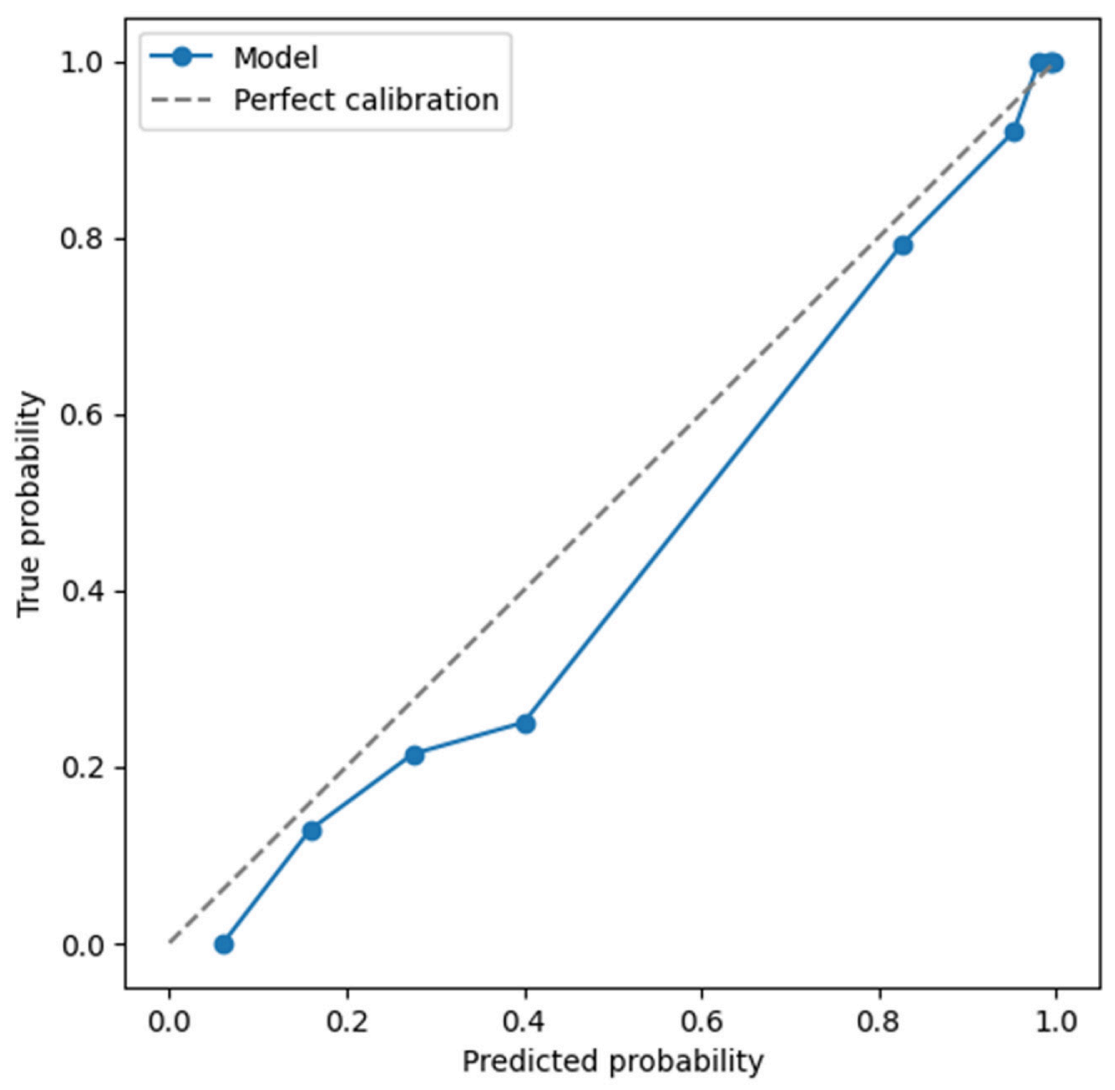
Calibration plot of the revised model in the external validation cohort.

The calibration plot (Figure 6) demonstrated good correspondence between predicted and observed probabilities across the range of predicted risks. The Brier score was 0.06, indicating good overall calibration and predictive performance.

## 4. Discussion

### 4.1. Validation and Refinement of the Model

In this follow-up study, we externally validated and thereafter refined the previously published diagnostic model for identifying high-grade lymphoma in children with cervical lymphadenopathy [17]. Our results demonstrate that, when applied to an independent cohort, the original scoring model performed within the 95% confidence intervals reported in the initial publication. This confirms the validity of the original model, even though baseline differences between the original and new dataset were observed. The most important difference, a higher proportion of patients with elevated LDH in the validation cohort, did not adversely affect the performance of the model.

In addition to validating the model, we refined it based on clinical feedback and systematic reassessment. A key strength of the updated model is its simplified structure. Several overlapping variables or variables with low diagnostic weight were removed without compromising diagnostic performance. Despite reassessing the model with an entirely new cohort and a new univariate analysis, no additional predictors emerged as statistically meaningful. This strengthens the validity of the original variable set and suggests that the principal predictors of high-grade lymphoma in this population are now well defined. The reduction in the number of predictors enhances the use of the model in clinical practice, particularly in busy general pediatric or emergency care settings where rapid decision making is required. Despite using fewer variables, the updated model demonstrated consistent diagnostic accuracy in the new cohort. Furthermore, the updated model demonstrated adequate calibration, indicating that predicted risks corresponded well with observed outcomes in the validation cohort.

In the updated model, TARC remains one of the most significant predictors, consistent with the original model. Further research is ongoing to define the role of TARC in the general pediatric setting, particularly in patients with a lower pre-test probability of malignancy and in settings outside tertiary oncology centers. Nevertheless, this study emphasizes the diagnostic potential of TARC in differentiating between malignant and benign lymphadenopathy as well. An important consideration for broader implementation is the availability of TARC testing. Although TARC is currently not routinely measured in all pediatric practices, its accessibility is expected to increase in the coming years. Beyond lymphoma diagnostics, TARC has emerged as a promising biomarker in other diseases, including atopic dermatitis and asthma [25,26,27]. As a result, testing infrastructure is becoming more widely available. Furthermore, the costs associated with TARC measurement are relatively low compared to many other specialized laboratory tests. Together, these developments may facilitate broader implementation of TARC-based diagnostic models in routine pediatric practice. However, ablation studies showed that removing TARC as a predictor results in a decrease in specificity from 88% to 86% indicating that TARC contributes to diagnostic performance, while also demonstrating that the model does not rely exclusively on TARC.

The implementation of age-adjusted LDH thresholds addressed an important biological limitation of the original model. LDH values are known to vary substantially with age, especially in younger children. The fixed cut-off used previously may have led to inaccurate risk estimation in this subgroup. Replacing the absolute threshold with an age-specific definition of elevated LDH improved discrimination without increasing the complexity of the model.

With these improvements, the updated model maintained high sensitivity, which is important for avoiding missed lymphoma cases, while showing a comparable specificity. The correct balance between sensitivity and specificity is essential, as earlier studies demonstrated that false-positive referrals may lead to unnecessary stress, biopsies, and healthcare visits for patients and their families [28].

Despite its high sensitivity and specificity, the revised model resulted in nine false negative and eight false positive cases in the new cohort. The false negative patients were mostly diagnosed with non-Hodgkin lymphoma. These patients typically presented with enlarged cervical lymph nodes and no other abnormalities. In two of these false negative patients, there were missing variables, which could have influenced the result of the model. In the false positive group, two patients were diagnosed with tuberculosis, one with systemic lupus erythematosus (SLE) and one with an underlying immunodeficiency. These are serious conditions, which can mimic the clinical presentation of advanced lymphoma.

### 4.2. Clinical Application and Implementation

A large proportion of variables in the model can be obtained through careful physical examination, for example, the location of the lymph node and hepatosplenomegaly. This highlights the importance of thorough clinical evaluation as the first step in the assessment of pediatric lymphadenopathy. However, the purpose of the model is to support structured decision making by integrating clinical findings with additional diagnostic information. While some patients with clear clinical signs of malignancy may already be identified based on physical examination alone, a considerable proportion of patients present with less specific or ambiguous findings. In these cases, the model provides added value by combining multiple clinical, radiological, and laboratory parameters to improve risk assessment. Therefore, in cases where clinical assessment alone does not lead to a clear conclusion, we advise performing additional diagnostic investigations, including basic laboratory testing (including LDH and TARC), chest X-Ray imaging, and ultrasound evaluation of the lymph nodes.

In clinical practice, the model is intended for children presenting with persistent or otherwise unexplained cervical lymphadenopathy in whom lymphoma is considered part of the differential diagnosis. Once the required data have been collected, the score can be calculated to support referral decisions. Children exceeding the referral threshold (32 points) should be discussed with or referred to a pediatric oncology center, whereas those with low scores may be managed locally according to clinical judgment and follow-up findings. For patients with intermediate or inconclusive risk profiles, the model should be interpreted alongside clinical expertise and shared decision making with the patient and their family.

### 4.3. The Previous Literature

To contextualize the current findings within the recent literature, studies published after the development of the original model were reviewed [10,13,18,19,21]. Several studies confirmed the importance of predictors already incorporated into our model. Age, B-symptoms, hepatosplenomegaly, supraclavicular lymphadenopathy, and mediastinal involvement were consistently associated with an increased risk of malignancy, while elevated ESR, LDH, uric acid, and pathological lymph nodes on ultrasound were also identified as relevant predictors. These findings closely overlap with the predictors identified in both the original and current cohorts, supporting the robustness of the selected variables.

Although several studies have applied multivariable regression techniques, few have developed clinically applicable prediction models. Haimi-Cohen et al. and Six et al. identified lymph node size as one of the strongest predictors of malignancy, while Isik et al. reported both lymph node size and supraclavicular involvement as independent predictors [6,9,14]. These findings are consistent with our observations that enlarged lymph nodes are more frequently observed in malignant disease. Consequently, lymph node size was evaluated as a potential additional predictor during model refinement. However, incorporation of a size-based variable did not improve overall model performance and was therefore not retained in the final model. This finding illustrates that predictors identified in univariate or multivariable analyses do not necessarily provide additional value once incorporated into a broader diagnostic model.

Compared with previously published multivariable models, the current nine-factor model combines information obtained from physical examination, laboratory investigations, and imaging studies into a single decision-support tool. Celenk et al. reported sensitivities of 77.9–84.0% and specificities of 61.5–67.9%, whereas Six et al. achieved a sensitivity of 100% using a lymph node size threshold of 2 cm, but at the cost of a specificity of only 54% [6,8]. On the other hand, Kamis et al. reported excellent specificity (99.6%) but limited sensitivity (60.7%). However, our model was specifically designed to maintain high sensitivity while preserving acceptable specificity, since we aim to avoid missed lymphoma diagnoses.

Another important distinction is that our model has undergone both external validation and subsequent refinement based on clinical feedback. Furthermore, age-adjusted LDH thresholds were introduced to account for physiological age-related variation in LDH levels. To our knowledge, previous prediction models have not incorporated age-specific LDH thresholds. Together with the reduction from 12 to 9 predictors, these modifications improved clinical usability while maintaining diagnostic performance.

### 4.4. Limitations and Future Directions

As in the original publication, this study has several limitations. Consistent with the findings from the previous publication, the updated model does not detect low-grade lymphomas such as nodular lymphocyte-predominant Hodgkin lymphoma (NLPHL). As previously argued, failing to identify such cases at first presentation may be clinically acceptable, given the low proliferation rate and good prognosis of this malignancy. Nonetheless, further research is needed to better identify risk factors specific to low-grade lymphoid malignancies and to develop complementary diagnostic strategies specific to this group.

Another methodological consideration concerns the handling of missing predictor values. Missing predictor values were coded as negative, consistent with the methodology used during development of the original model. This approach was based on the clinical assumption that diagnostic investigations are less likely to be performed when suspicion of abnormalities is low. Because this assumption might introduce bias, sensitivity analyses were performed using alternative missing-data strategies. Reassuringly, model performance remained largely unchanged when missing values were handled as a separate category or randomly assigned. Although complete-case analysis resulted in a lower specificity, this is likely explained by the higher proportion of missing data among patients with benign disease, resulting in a more selective study population. Taken together, these findings support the validity of the original missing-data strategy and indicate that missing values did not substantially influence model performance.

The updated model has so far only been validated in a specific tertiary care setting with a high malignancy rate (62.4%). Furthermore, both the development and validation cohorts originated from the same tertiary referral center. Therefore, the present study should be regarded as a temporal rather than a geographic external validation. While this supports the robustness of the model over time, patient characteristics, referral patterns, and diagnostic pathways are likely to differ from those in community hospitals and general pediatric practice. The positive predictive value of the model would likely be lower in general pediatric settings, where the prevalence of malignancy among children presenting with cervical lymphadenopathy is substantially lower [5,10,11,15]. The model should therefore be interpreted primarily as a tool for risk stratification rather than definitive diagnosis. Nevertheless, the intended role of the model is to support referral and further diagnostic decision making. A lower positive predictive value in general practice may be acceptable, given that the model maintains high sensitivity and safely identifies children who require further evaluation.

Additionally, prospective validation in other healthcare settings is necessary to confirm the stability and flexibility of the model and verify its usefulness in routine decision making. Future research should focus on the model’s prospective multicenter implementation, and assessment of its impact on referral patterns, diagnostic workload, and patient-centered outcomes. Our research group is currently conducting such a prospective study, with the goal of validating the model in a general pediatric setting (International Clinical Trials Registry Platform NL-OMON57739).

## 5. Conclusions

In conclusion, our updated diagnostic nine-factor model maintains the high diagnostic performance of the original version while having a reduced number of variables. These findings support the implementation of the refined model as a practical decision-support tool for early identification of children at risk of high-grade lymphoma.

## Figures and Tables

**Table 1:** Univariate analysis of variables analyzed as prediction factors for malignancy.

Variable	Malignant Group (*n* =159) Median (IQR) or *n* (%)	m	Benign Group (*n* = 96) Median (IQR) or *n* (%)	m	*p*-Value	95% CI
General						
Age (years)	15 (12–16)	-	11 (5–12)	-	<0.001	-
Hepatosplenomegaly	31 (19.5)	-	10 (10.5)	-	0.077	0.92–4.95
Radiology						
Unilateral/bilateral lymph nodes	114 (71.7)/ 45 (28.3)	-	44 (46.3)/ 51 (53.7)	-	<0.001	1.7–5.2
Pathological lymph nodes on ultrasound	156 (100)	3	54 (59.3)	5	<0.001	26.0–∞
>3 cervical levels involved	54 (35.8)	8	3 (3.2)	1	<0.001	5.2–87.5
Level V involvement	125 (83.3)	9	14 (15.1)	3	<0.001	13.2–61.8
Supraclavicular lymph node	145 (91.8)	1	17 (17.7)	0	<0.001	22.6–121.2
Infraclavicular lymph node	51 (33.1)	5	1 (1)	0	<0.001	7.7–1895.4
Mediastinal lymphadenopathy	137 (86.2)	0	4 (4.5)	7	<0.001	42.2–533.1
Enlarged mediastinum	110 (69.6)	1	4 (4.2)	0	<0.001	18.0–205.8
Short axis largest lymph node (cm)	1.8 (1.4–2.2)	16	1.4 (1.1–2.0)	8	<0.001	-
Short axis > 2 cm	47 (32.9)	16	19 (21.6)	8	0.073	0.93–3.49
Number of body regions involved	5 (3–6)	-	1 (1–2)	-	<0.001	-
>3 body regions involved	113 (71.1)	-	5 (5.2)	-	<0.001	16.6–174.4
Laboratory						
TARC (pg/mL)	6026 (1232–10.000)	4	85 (72–378)	30	<0.001	-
TARC > 850 pg/mL	119 (76.8)	4	2 (3)	30	<0.001	25.3–930.4
LDH > reference value Maxima	79 (51.3)	5	30 (34.5)	8	0.015	0.28–0.89
Neutrophils > 6 × 10^3^/mm^3^	98 (64.2)	7	21 (23.1)	5	<0.001	3.2–11.3
CRP (ug/mL)	42 (13–91)	44	4 (1–11)	24	<0.001	-
ESR> 20 mm/h	103 (73.6)	19	27 (32.1)	12	<0.001	3.1–11.1

Abbreviations: m—missing; IQR—interquartile range; TARC—thymus and activation-regulated chemokine, LDH—lactate dehydrogenase, CRP—C-reactive protein; ESR—erythrocyte sedimentation rate; Máxima—Princes Máxima Center.

**Table 2:** Factors included in the new model with their definitions and weighting factors.

Variable	Definition	Weighting Factor
Mediastinum/hilum involved	Enlarged mediastinum or hilar lymphadenopathy on chest X-ray or chest CT	18.5
TARC > 850 pg/mL	TARC above 850 pg/mL at first presentation	18
Pathological lymph nodes on ultrasound	Presence of pathological lymph nodes as described by the radiologist	12
Cervical levels > 3	Presence of at least one pathological lymph node in three or more cervical levels on imaging	12
Infraclavicular lymph nodes	Presence of at least one palpable or radiologically confirmed infraclavicular lymph node	10
Body regions > 3	Presence of pathological lymph node or mass in more than three body regions. Specification of the different body regions is available in the Supplementary Material of Zijtregtop et al. [17].	9
Supraclavicular lymph nodes	Presence of at least one palpable or radiologically confirmed supraclavicular lymph node	7
LDH > Princess Máxima Center cut-off	LDH above the age-adjusted upper limit of normal according to reference values (Table S4)	7
Hepatosplenomegaly	Liver and/or spleen enlargement documented during physical examination or imaging	5

Abbreviations: TARC—thymus and activation-regulated chemokine; LDH—lactate dehydrogenase.

## Data Availability

The data presented in this study are available on request from the corresponding author. The data are not publicly available due to ethical considerations. The codes of the machine learning model are available on request from the corresponding author.

## Supplementary Materials

The following supporting information can be downloaded at: https://www.mdpi.com/article/10.3390/cancers18152407/s1, Table S1: Baseline characteristics of old and new datasets; Table S2: Detailed comparison of characteristics of old and new datasets; Table S3: LDH subgroup analysis; Table S4: Age-adjusted LDH reference values used by the Princess Máxima Center; Table S5: Additional information on misclassified patients; Table S6: Sensitivity analysis of different approaches for handling missing predictor values; Table S7: Ablation study results; Table S8: Performance of both the old and the new models.

## Abbreviations

The following abbreviations are used in this manuscript:

ALCL	Anaplastic large-cell lymphoma
AUC	Area under the curve
BL	Burkitt lymphoma
B-LBL	B-cell lymphoblastic lymphoma
cHL	Classical Hodgkin lymphoma
CRP	C-reactive protein
DLBCL	Diffuse large-B-cell lymphoma
ESR	Erythrocyte sedimentation rate
IQR	Interquartile range
LDH	Lactate dehydrogenase
PMBCL	Primary mediastinal large-B-cell lymphoma
Máxima	Princes Máxima Center
NHL	Non-Hodgkin lymphoma
NLPHL	Nodular lymphocyte-predominant Hodgkin lymphoma
PTGC	Progressive transformation of germinal centers
PTLD	Post-transplant lymphoproliferative disorder
ROC	Receiver operating characteristic
SLE	Systemic lupus erythematosus
TARC	Thymus and activation-regulated chemokine
T-LBL	T-cell lymphoblastic lymphoma
