# Supplementary material for: External Validation and Feedback-Driven Improvement of the Diagnostic Model for Pediatric Cervical Lymphadenopathy
Source: Cancers (Basel). 2026 Jul 26;18(15):2407. doi: 10.3390/cancers18152407 (PMC13464063; doi:10.3390/cancers18152407)
Supplement: Supplementary file 1 [file cancers-18-02407-s001.zip › cancers-4403050-supplementary.pdf]

Supplementary Materials - Improving the Diagnostic Model for Pediatric Cervical Lymphadenopathy: External Validation and Feedback-Driven Model Updating

Table S1. Baseline characteristics of old and new datasets

Variable	**Old dataset (N=182)** Median (IQR) or n (%)	**New dataset (N=255)** Median (IQR) or n (%)	**Difference between old and new dataset (P-value)**
**General**			
Age (years)	13 (9-15)	14 (10-16)	0.025
Gender (female)	82 (45.1)	117 (45.9)	0.922
**Diagnosis**			
Malignant diagnosis	115 (63.2)	158 (62.4)	0.920
**Subclassification malignant diagnoses**			0.696
Hodgkin lymphoma	90	124	
Non Hodgkin lymphoma			
ALCL	5	10	
BL	5	4	
DLBCL	3	5	
PMBCL	1	3	
T-LBL	8	12	
B-LBL	2	0	
Other malignancy	1	0	
**Subclassification benign diagnoses**			0.564
Reactive/infectious lymphadenopathy	48	76	
Immunological disorder	5	2	
PTGC	4	1	

Morbus Castleman	2	2
Rheumatological disorder	2	3
Branchiogenic cyst	3	5
Venous malformation	1	1
Lymphangioma	1	2
Dermoid cysts	1	0
Biphenotypic branchioma	0	1
Pleomorphic adenoma	0	1
Thoracic duct cyst	0	1
Kikuchi-Fujimoto disease	0	1

Abbreviations: ALCL – anaplastic large cell lymphoma; PMBCL – primary mediastinal large B-cell lymphoma; DLBCL – diffuse large B-cell lymphoma; BL – Burkitt lymphoma; T-LBL – T-cell lymphoblastic lymphoma; B-LBL – B-cell lymphoblastic lymphoma; PTGC – progressive transformation of germinal centers.

Table S2: Detailed comparison of characteristics of old and new datasets

**Variable**	**Old dataset (N=182)** Median (IQR) or n (%)	**m**	**New dataset (N=255)** Median (IQR) or n (%)	**m**	**P-value**	**95% CI**
			**General**			
Age (years)	13 (9-15)	0	14 (10-16)	0	0.025	-
Gender (female)	82 (45.1)	0	117 (45.9)	0	0.922	0.65 - 1.45
Hepatosplenomegaly	48 (28.9)	16	41 (16.1)	2	0.002	0.29 - 0.78
			**Radiology**			
Pathological lymph nodes ultrasound	132 (77.6)	12	210 (85)	8	0.069	0.96 - 2.79
>3 cervical levels involved	40 (24)	15	57 (23.2)	9	0.906	0.59 - 1.57
Level V involvement	83 (49.7)	15	139 (57.2)	12	0.158	0.89 - 2.05

Supraclavicular lymph node	116 (63.7)	0	162 (63.8)	1	1	0.66 - 1.52
Infraclavicular lymph node	39 (21.4)	0	52 (20.8)	5	0.905	0.59 - 1.58
Mediastinal or hilar lymphadenopathy	95 (52.2)	0	141 (56.9)	7	0.378	0.81 - 1.80
Enlarged mediastinum	66 (37.1)	4	114 (44.9)	1	0.113	0.92 - 2.09
Short axis > 2 cm	34 (20.5)	16	67 (28.9)	23	0.062	0.96 - 2.61
>3 body regions involved	86 (47.3)	0	118 (46.3)	0	0.846	0.65 - 1.43
			**Laboratory**			
TARC > 850 pg/ml	75 (51.7)	37	121 (54.8)	34	0.593	0.73 - 1.76
LDH > 260 U/l	84 (49.7)	12	133 (55.2)	14	0.317	0.82 - 1.86
LDH > reference value Máxima	54 (31.8)	12	110 (45.6)	14	0.006	1.17 - 2.78
Neutrophils > 6 x 10^3/mm3	67 (37.2)	2	119 (49.2)	13	0.017	1.08 - 2.47
ESR> 20 mm/h	98 (57)	10	129 (57.8)	32	0.918	0.68 - 1.58

Abbreviations: TARC - thymus and activation-regulated chemokine, LDH – lactate dehydrogenase, ESR – erythrocyte sedimentation rate

Table S3: LDH subgroup analysis

LDH subgroups

Variable	HL (N =119)	NHL (N = 33)	Benign (N = 87)	P-value
LDH (U/l) - median	274	312	259	0.040*

* Kruskal-Wallis test

Results by LDH subgroup

Benign vs cHL: p-value 0.490*
Benign vs NHL: p-value 0.005*
cHL vs NHL: p-value: 0.010*
* Mann-Whitney U test

Table S4: Age-adjusted LDH reference values used by the Princess Máxima Center

Age	LDH threshold (U/l)
Up to 1 month	0-765
1 month – 1 year	0-450
1 year – 3 years	0-395
3 years – 6 years	0-345
6 years – 15 years	0-325
15 years – 18 years	0-250

Table S5: Additional information on misclassified patients

Patient	True diagnosis	Biopsy	Model prediction	Mediastinum/ Hilum involved	TARC >850	Pathological lymph nodes on US	Cervical levels >3	Infra-clavicular nodes	Body regions >3	Supra-clavicular nodes	LDH elevated	HSM	Scored points
FN1	ALCL	Yes	Benign	No	No	Yes	No	No	Yes	No	No	No	21
FN2	ALCL	Yes	Benign	No	No	Yes	No	No	No	Yes	No	No	19
FN3	DLBCL	Yes	Benign	No	Missing	Yes	No	No	No	No	No	No	12
FN4	BL	Yes	Benign	No	No	Yes	No	No	No	No	No	No	12
FN5	DLBCL	Yes	Benign	No	No	Yes	No	No	No	No	No	No	12
FN6	ALCL	Yes	Benign	No	No	Yes	No	No	No	Yes	No	No	19
FN7	DLBCL	Yes	Benign	No	No	Yes	No	No	No	No	No	No	12
FN8	BL	Yes	Benign	No	No	Yes	No	No	No	No	Yes	No	19
FN9	cHL	Yes	Benign	No	No	Yes	Yes	No	No	Yes	Missing	No	31
FP1	Toxoplasmosis	Yes	Malignant	No	No	Yes	No	No	Yes	Yes	Yes	Yes	40
FP2	EBV	No	Malignant	No	Missing	Yes	Yes	No	No	Yes	Yes	No	38

FP3	Tuberculosis	No	Malignant	Yes	No	Yes	No	No	Yes	Yes	No	Yes	51.5
FP4	SLE	No	Malignant	Yes	Missing	Yes	No	No	No	No	No	Yes	35.5
FP5	Immunodeficiency	Yes	Malignant	Yes	Yes	Missing	No	No	Yes	Yes	No	Yes	57.5
FP6	Infectious	Yes	Malignant	No	No	Yes	No	No	Yes	Yes	Yes	No	35
FP7	Tuberculosis	Yes	Malignant	Yes	No	Yes	Missing	No	Yes	Yes	No	No	46.5
FP8	Retropharyngeal abscess	Yes	Malignant	Yes	Missing	Yes	Yes	No	No	No	No	No	42.5

Abbreviations: TARC - thymus and activation-regulated chemokine; US – ultrasound; LDH – lactate dehydrogenase; HSM – Hepatosplenomegaly; ALCL – anaplastic large cell lymphoma; DLBCL – diffuse large B-cell lymphoma; BL – Burkitt lymphoma; cHL – classical Hodgkin lymphoma; EBV – Epstein Barr virus; SLE – systemic lupus erythematosus

Table S6: Sensitivity analysis of different approaches for handling missing predictor values

Missing data strategy	Sensitivity	Specificity	Interpretation
Missing values coded as negative (original model)	93%	88%	Original model
Complete-case analysis	96%	79%	Lower specificity due to exclusion of patients with missing data, predominantly benign cases
Missing values as separate category	94%	86%	Comparable performance
Random assignment of missing values	94%	87%	Comparable performance

Table S7: Ablation study results

Model version	Sensitivity	Specificity	AUC	False positive count	False negative count
Original version (2023)	93%	88%	0.96 (0.91-0.99)	7/171	8/171
Excluding '>3 cervical lymph nodes'	92%	85%	0.95 (0.91-0.98)	8/171	10/171
Excluding 'mediastinum/hilum involved'	95%	87%	0.96 (0.92-0.99)	5/171	9/171
Excluding 'TARC > 850 pg/ml'	93%	86%	0.95 (0.91-0.98)	7/171	9/171
Excluding 'pathological lymph nodes on US'	91%	88%	0.95 (0.91-0.99)	9/171	8/171
Excluding 'enlarged mediastinum'	93%	88%	0.96 (0.91-0.99)	7/171	8/171
Excluding 'infraclavicular lymph node'	93%	88%	0.95 (0.91-0.99)	7/171	8/171
Excluding '>3 body regions'	94%	90%	0.96 (0.92-0.99)	6/171	7/171
Excluding 'level V lymph node'	96%	90%	0.95 (0.91-0.99)	4/171	7/171
Excluding 'supraclavicular lymph node'	96%	90%	0.96 (0.91-0.99)	4/171	7/171
Excluding 'Hepatosplenomegaly'	94%	90%	0.96 (0.91-0.99)	6/171	7/171
Excluding 'LDH >260 U/l'	94%	88%	0.96 (0.91-0.99)	6/171	8/171
Excluding 'neutrophils >$6x10^3/mm^3$'	93%	90%	0.96 (0.91-0.99)	7/171	7/171
Excluding 'neutrophils >$6x10^3/mm^3$' and 'enlarged mediastinum'	93%	90%	0.96 (0.91-0.99)	7/171	7/171
Excluding 'neutrophils >$6x10^3/mm^3$' and 'mediastinum/hilum involved'	96%	87%	0.96 (0.91-0.99)	4/171	9/171
Excluding 'neutrophils >$6x10^3/mm^3$', 'enlarged mediastinum' and 'level V lymph node'	95%	91%	0.95 (0.91-0.99)	5/171	6/171
Excluding 'neutrophils >$6x10^3/mm^3$', 'enlarged mediastinum' and 'supraclavicular lymph node'	96%	90%	0.96 (0.91-0.99)	4/171	7/171

NOTE. All performances are tested on the development cohort

Abbreviations: TARC - thymus and activation-regulated chemokine; US – ultrasound; LDH – lactate dehydrogenase; AUC – area under the curve

Table S8: Performance of both the old and the new models

Performance metric	Old model		New model	
	Development cohort	External validation cohort	Development cohort	External validation cohort
Sensitivity	93%	94%	95%	94%
Specificity	88%	91%	90%	91%
AUC (95% CI)	0.96 (0.91-0.99)	0.98 (0.96-0.99)	0.95 (0.90-0.99)	0.98 (0.96-0.99)
False negative count	7/171	9/255	5/171	9/255
False positive count	8/171	8/255	7/171	8/255

Abbreviations: AUC – area under the curve; CI – confidence interval
